# Tanshinone I exerts cardiovascular protective effects in vivo and in vitro through inhibiting necroptosis via Akt/Nrf2 signaling pathway

**DOI:** 10.1186/s13020-021-00458-7

**Published:** 2021-06-28

**Authors:** Youqiong Zhuo, Renyikun Yuan, Xinxin Chen, Jia He, Yangling Chen, Chenwei Zhang, Kaili Sun, Shilin Yang, Zhenjie Liu, Hongwei Gao

**Affiliations:** 1 College of Pharmacy, Guangxi University of Chinese Medicine, Nanning, 530000 China; 2 Guangxi Engineering Technology Research Center of Advantage Chinese Patent Drug and Ethnic Drug Development, Nanning, 530200 China; 3 State Key Laboratory of Innovative Drug and Efficient Energy-Saving Pharmaceutical Equipment, Jiangxi University of Traditional Chinese Medicine, Nanchang, 330004 China

**Keywords:** Tanshinone I, Oxidative stress, Necroptosis, Myocardial ischemia reperfusion, RIP1/RIP3/MLKL, Akt/Nrf2

## Abstract

**Background:**

Tanshinone I (TI) is a primary component of *Salvia miltiorrhiza* Bunge (Danshen), which confers a favorable role in a variety of pharmacological activities including cardiovascular protection. However, the exact mechanism of the cardiovascular protection activity of TI remains to be illustrated. In this study, the cardiovascular protective effect and its mechanism of TI were investigated.

**Methods:**

In this study, tert-butyl hydroperoxide (t-BHP)-stimulated H9c2 cells model was employed to investigate the protective effect in vitro. The cell viability was determined by 3-(4, 5-dimethylthiazol-2-yl)-2, 5-diphenyl tetrazolium bromide (MTT) assay and lactate dehydrogenase (LDH) kit. The reactive-oxygen-species (ROS) level and mitochondrial membrane potential (MMP) were investigated by the flow cytometry and JC-1 assay, respectively. While in vivo experiment, the cardiovascular protective effect of TI was determined by using myocardial ischemia–reperfusion (MI/R) model including hematoxylin–eosin (H&E) staining assay and determination of superoxide dismutase (SOD) and malondialdehyde (MDA). Tumor necrosis factor-α (TNF-α) and interleukin-6 (IL-6) release were detected by Enzyme-linked immunosorbent assay (ELISA). Receptor interacting protein kinase 1 (RIP1), receptor interacting protein kinase 3 (RIP3), receptor interacting protein kinase 3 (MLKL), protein kinase B (Akt), Nuclear factor erythroid 2 related factor 2 (Nrf2), Heme oxygenase-1 (HO-1) and NAD(P)H: quinone oxidoreductase-1 (NQO-1) were determined by western blotting.

**Results:**

Our data demonstrated that TI pretreatment attenuated t-BHP and MI/R injury-induced necroptosis by inhibiting the expression of p-RIP1, p-RIP3, and p-MLKL. TI activated the Akt/Nrf2 pathway to promote the expression of antioxidant-related proteins such as phosphorylation of Akt, nuclear factor erythroid 2 related factor 2 (Nrf2), quinone oxidoreductase-1 (NQO-1) and heme oxygenase-1 (HO-1) expression in t-BHP-stimulated H9c2 cells. TI relieved oxidative stress by mitigating ROS generation and reversing MMP loss. In vivo experiment, TI made electrocardiograph (ECG) recovery better and lessened the degree of myocardial tissue damage. The counts of white blood cell (WBC), neutrophil (Neu), lymphocyte (Lym), and the release of TNF-α and IL-6 were reversed by TI treatment. SOD level was increased, while MDA level was decreased by TI treatment.

**Conclusion:**

Collectively, our findings indicated that TI exerted cardiovascular protective activities in vitro and in vivo through suppressing RIP1/RIP3/MLKL and activating Akt/Nrf2 signaling pathways, which could be developed into a cardiovascular protective agent.
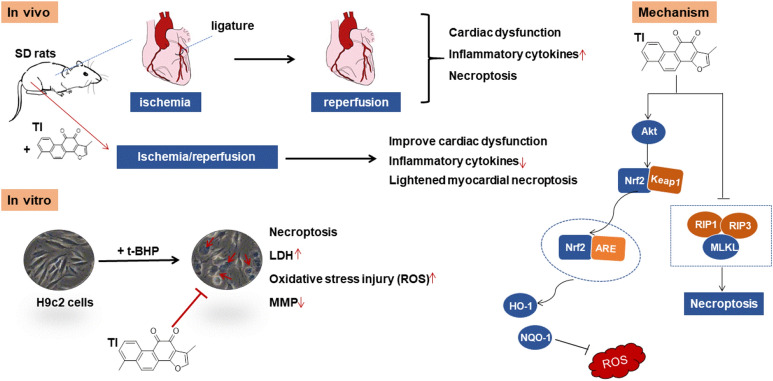

**Supplementary Information:**

The online version contains supplementary material available at 10.1186/s13020-021-00458-7.

## Background

The incidence and mortality of cardiovascular diseases have been increasing in recent years [1], and myocardial ischemia represents a major contributor to cardiovascular diseases [2]. Myocardial ischemia refers to the gradual decrease in blood perfusion to the heart, resulting in a reduction in oxygen supply to the heart and insufficient energy metabolism of the heart muscle, which cannot guarantee normal and regular work of the heart [3]. At present, the main therapeutic strategy is to restore the blood supply to the myocardial ischemia site in multiple ways. MI/R, one of the most representative models, will also bring about a series of metabolic disorders, dysfunction, and structural destruction of tissues and organs, such as arrhythmias, enlarged infarcted area, ventricular dysfunction, and energy metabolism disorders, and other additional reperfusion damage [4, 5]. In addition, MI/R injury is a common and inevitable problem in coronary interventional therapy. In addition to intervention to solve the heart's large vessel problems, drug therapy has become the main method to improve this injury. Traditional Chinese medicine owns a variety of advantages and characteristics such as multi-component, multi-target, multi-path, and low economic cost [6, 7]. Therefore, it is of great significance to explore effective constituent to improve MI/R injury.

MI/R injury is the key to poor prognosis of patients [5]. The onset of this damage is related to calcium overload, inflammatory response, energy metabolism disorder and oxygen-free radicals. And it can cause damage to the myocardium [4], promote the occurrence of myocardial remodeling, increase myocardial infarction area and facilitate the release of various inflammatory factors [8, 9] and oxygen free radicals [10]. Among them, the production of oxygen free radicals is closely related to MI/R injury. In patients with myocardial ischemia, the content of free radical scavenging agents will be reduced [11], leading to the rise of free radicals, which not only motivates cell damage but also accelerates cell death [12]. Accordingly, in the treatment of patients, effective scavenging of oxygen free radicals and cardiomyocytes’ damage can be reduced by inhibition of the generation of oxygen free radicals.

Necroptosis, a form of necrosis, can be controlled, in which dead cells rupture and liberate components inside the cells incurring cell death. In contrast to apoptosis, necroptosis leads to fracture of the plasma membrane, thus cell contents overflow, bringing about inflammation and the triggering of the immune system. Necroptosis signals need the involvement of RIP1, RIP3, and MLKL kinase [13]. There is growing literature that programmed necrosis occupies a crucial part in MI/R [14]. Therefore, suppression of necroptosis is an alternative strategy for the treatment of cardiovascular diseases [15]. This study will start with RIP1/RIP3/MLKL signaling pathway to explore the relationship between programmed necrosis and MI/R, aiming to provide a new perspective of current research for the prevention and treatment of MI/R from the perspective of “programmed necrosis intervention” [16–18].

A previous study indicated that ROS always leads to cells necroptosis. Akt/Nrf2 pathway is crucial to modulate ROS generation [19]. Akt participates in the positive regulation of Nrf2 antioxidant. Then Nrf2 activated further separates from Keap1, entering into the nucleus to form dimers with Maf, recognizing and binding to the binding sites on ARE which will induce the transcription of NQO-1 as well as HO-1 to reduce oxidative stress injury [19, 20]. Accordingly, the regulation of Akt/Nrf2 signaling pathway to reduce ROS generation-induced necroptosis will be an effective therapeutic strategy for the treatment of cardiovascular diseases.

**Fig. 1:**
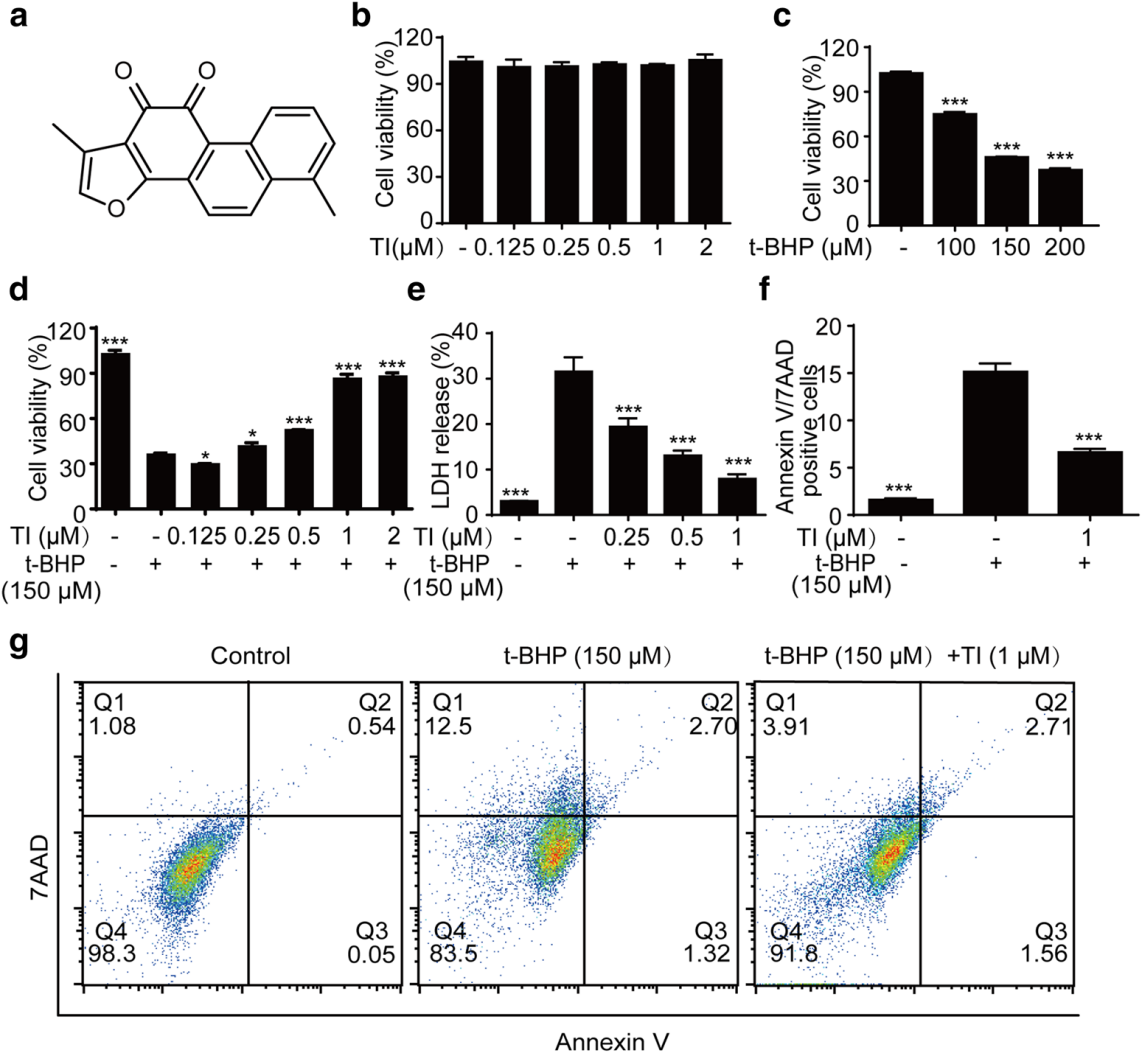
Tanshinone I (TI) attenuated t-BHP-induced cell death in H9c2 cells. **a** The chemical structure of TI. **b, c** H9c2 cells were treated with TI or t-BHP for a series of concentrations for 12 h or 10 h respectively, and cell viability was both tested by MTT. Cells were administrated with t-BHP for 10 h after treated with TI for 2 h. **d** Employed MTT method to detect the viability of the cells. **e** Employed LDH kit to monitor the LDH level. **f–g** Cells pretreated with TI (1 μM) for 2 h before co-incubation with t-BHP (150 μM) for 6 h, were then stained with Annexin V-PE /7-AAD and detected by flow cytometry. n = 3. ***p < 0.001 *vs*. t-BHP group

*Salvia miltiorrhiza* Bunge (Danshen) is a traditional Chinese medicine that has widely incorporated into therapy for cardiovascular diseases through invigorating blood circulation and dispersing blood stasis [21]. Tanshinone I (TI) (Fig. 1a), a fat-soluble compound, is a major ingredience in Danshen [22]. However, the mechanism of cardiovascular protection of TI has not been revealed. Therefore, exploring the pharmacological effects of TI also provides new ideas for the treatment of cardiovascular diseases by Danshen. In this study, using t-BHP-stimulated H9c2 cells and MI/R SD rat model, we investigated TI’s cardiovascular protective effect and mechanism in vitro and in vivo.

## Materials and methods

### Chemical and reagents

Tanshinone I was purchased from Chengdu Pufei De Biotech Co., Ltd, (Chengdu, China), while its purity (over 98%) was measured by HPLC. Verapamil hydrochloride was selected and bought from a Shanghai Macklin Biochemical Technology Co., Ltd, (Shanghai, China). 2′, 7′-Dichlorodihydrofluorescein diacetate (DCFH_2_-DA), t-BHP, Dimethyl sulfoxide (DMSO), MTT and Necrostatin-1 (Nec-1) were obtained from Sigma-Aldrich (St. Louis, MO, USA). Double staining kit (7-AAD & Annexin V-PE) was bought from BD Pharmingen™ (Becton–Dickinson, NJ, USA). From Life Technologies/Gibco Laboratories (Grand Island, NY, USA), fetal bovine serum (FBS), as well as dulbecco’s modified eagle medium (DMEM) were bought. JC-1 kit and LDH assay kit were obtained from Beyotime (Shanghai, China). From Thermo Fisher Scientific (Waltham, MA, USA), BCA protein detection kit was obtained. ELISA kits from Neobioscience (Shenzhen, China). MDA, as well as SOD detection kits, were purchased on selection from Nanjing Jiancheng Bioengineering Institute (Nanjing, China). Antibodies: p-RIP1 (AF7088), p-RIP3 (AF7443), p-MLKL (AF7420), RIP1(AF7877), and RIP3(AF7942) were purchased from Affinity Biosciences (Cincinnati, OH, USA), GAPDH (#5174), p-Akt (#4060), Akt (#4691), HO-1 (#70081), and secondary antibodies from Cell Signaling Technology (Beverly, MA, USA). MLKL (ab196436), Nrf2 (ab31163) and NQO1 (ab80588) were acquired from Abcam (Cambs, UK).

### Cell culture

H9c2 cells, purchased from the Cell Bank of the Chinese Academy of Sciences (Shanghai, China), were placed in DMEM medium (10% FBS and 1% Penicillin/Streptomycin) for cultivation in an incubator containing 5% CO_2_ with a specified temperature of 37 °C.

### Cell viability assay

4.0 × 10^3^ cells per well were cultured in 96-well plates overnight, which were treated with TI (0.125, 0.25, 0.5, 1, and 2 μM), Nec-1 (20, 40, 80, and 100 μM) or t-BHP (50, 100, and 150 μM) for 12 h, 12 h and 10 h subsequently. Our study employed MTT to detect the viability of cells and monitored the absorbance with a microplate reader (BioTek, Winkowski, VT, USA) at a wavelength of 570 nm.

### Apoptosis assay

H9c2 cells were cultured in 6-well plates (1.5 × 10^5^ cells per well). Afterwards, H9c2 cells were pretreated with TI (1 μM) for 2 h and treated with t-BHP (150 μM) for 6 h. After collection, cells were stained with Annexin V-PE/7-AAD and were detected by flow cytometry (Becton–Dickinson, NJ, USA).

### Measurement of lactate dehydrogenase (LDH) release

Inoculated on 96-well plates (4 × 10^3^ per well) overnight and pretreated with TI (0.25, 0.5, and 1 μM) for 2 h, H9c2 cells were co-cultured with t-BHP (150 μM) for 10 h. Medium was collected to measure LDH level as per the manufacturer’s instruction of LDH. Using a microplate reader (490 nm wavelength), the absorbance was determined.

### Intracellular reactive oxygen species (ROS) detection

H9c2 cells were seeded into 12-well plates (7.0 × 10^4^ cells per well) overnight and treated with t-BHP (150 μM) for a different time, respectively, which subsequently were co-cultured with TI (0.25, 0.5, and 1 μM) for 2 h. Then cells were labeled with DCFH_2_-DA (1 μM, 0.5 h), which were monitored by flow cytometry at the FITC channel. The images were captured by a fluorescence microscope (Leica, Wetzlar, Germany) [23].

### Mitochondrial membrane potential (MMP)’s detection assay

Based on our anterior study [24, 25], cells were cultured in 96-well plates (4.0 × 10^3^ cells in each well) overnight. After that, the cells were exposed to TI (1 μM) for 2 h and cultured with t-BHP (150 μM) up to 4 h. For measuring the MMP, JC-1 (5 µg/mL) was co-cultured for 30 min and relevant images were captured by a fluorescence microscope.

### Animal experiments and ethical statement

The study was authorized by the Experimental Animal Management Ethics Committee of Guangxi University of Traditional Chinese Medicine (Approval Document No. SYXK-GUI-2019-0001). As per the “Guidelines for the Care and Use of Laboratory Animals in Guangxi University of Traditional Chinese Medicine”, all animals have received humane care. Healthy SD rats (male, 220–250 g) were purchased from Hunan Saike Jingda Experimental Animal Co. Ltd (Changsha, China) and acclimated for one week. Under normative SPF (specific pathogen-free) circumstances, all animals were housed and had free access to water as well as food with a befitting and controllable humidity (50%) and temperature (25 °C).

Conventionally, the animals were divided into the sham-operated group, MI/R group, TI-L group (10 mg/kg), TI-H group (20 mg/kg), and positive drug Verapamil hydrochloride (Ver) group (20 mg/kg) at random and each group consisted of 15 rats. The rats were given pre-administration for one week before modeling. The rats were intraperitoneally injected with TI once a day while the sham operation group and MI/R group were given the uniform amount of normal saline at the corresponding time.

### Construction of MI/R SD rat model

The MI/R rats were operated by transient myocardial ischemia for 0.5 h and reperfusion for 2 h therewith. In short, the experimental rats were narcotized by intraperitoneal injection of 10% chloral hydrate (4 mL/kg). These rats were concatenated to a rodent ventilator (respiratory rate of 58–70 breaths/min, respiratory ratio of 5:4, tidal volume of 6–7 mL/time). The third and fourth ribs were cut open, leaving the heart nearly entirely exposed. Then 6–0 silk suture was used to ligature LAD (the left anterior descending coronary artery) at the distal 1/3. The heart grew grey promptly. The ligation was disentangled after occlusion for 30 min, the heart going through reperfusion for 2 h. The sham-operated group underwent the identical operation and was penetrated in absence of ligation. ECG changes were recorded throughout the whole experiment by BL-420N biological signal acquisition and analysis system (Chengdu, China).

### Histopathology examination

Fixed with 4% paraformaldehyde and dehydrated with alcohol gradient, the heart tissue samples were then processed with xylene transparently and paraffin-embedded. Hematoxylin and eosin (H&E) staining were employed after the samples were sectioned. Ultimately, with an optical microscope (UOP, DSZ5000X, China), the study observed the pathological changes of the tissue.

### Hematology analysis

Blood was collected from the abdominal aorta of rats. WBC, Neu, and Lym count from blood were determined by an auto hematology analyzer (Mindray, Shenzhen, China).

### Determination of SOD, MDA, TNF-α, IL-6

With a tissue grinder (Tianjin, China), SD rats’ heart tissue was homogenized. The samples were centrifuged. Their supernatant was collected immediately and stored at − 80 °C. As per the manufacturer’s instructions, the SOD, MDA examined by corresponding kits, TNF-α and IL-6 levels were detected by ELISA kits.

### Western blotting analysis

H9c2 cells were seeded into a dish overnight. TI (0.25, 0.5, and 1 μM) was pretreated for 2 h and co-cultured with t-BHP induction for 4 h. The study employed RIPA (1% PMSF and 1% cocktail) to extract total proteins of cells as well as the heart tissue. As per the manufacturer’s instruction, the concentration of the protein was measured by the BCA protein kit [25]. Through 10% or 12% SDS-PAGE gels, the denatured protein was separated, which was transferred to polyvinylidene fluoride (PVDF) membrane (Millipore, Billerica, MA, USA). After 5% skim milk blocking the PVDF membrane up to 2 h, incubate the membrane with 1:1000 primary antibodies (p-RIP1, p-RIP3, p-MLKL, RIP1, RIP3, MLKL, p-Akt, Akt, Nrf2, HO-1, NQO-1, GAPDH) at 4 °C for over 12 h. The membrane was exposed to ChemiDoc™ MP Imaging System (Bio-Rad, Hercules, CA, USA) after washing with TBST and incubating with secondary antibody (Anti-rabbit IgG, HRP-linked Antibody 1:5000) for 2 h at room temperature, of which GAPDH was the specified house-keeping protein.

### Statistical analysis

Dates were presented as means ± SD. All experiments were repeated at least three times. Dates were normally distributed and GraphPad Prism 6.0 software was used to perform one-way-ANOVA or Student’s *t*-test. When p < 0.05, results were considered to be statistically significant.

## Results

### TI exerted protective effects on t-BHP-stimulated H9c2 cells

The cytotoxicity of TI was detected by the MTT method. The results indicated that TI (0.125, 0.25, 0.5, 1, and 2 μM) displayed no significant toxicity in H9c2 cells as exhibited in Fig. 1b. In contrast with the control group, t-BHP (100, 150, and 200 μM) reduced cell viability to a diverse degree. t-BHP (150 μM) was selected as the befitting and conclusive concentration to conduct the following experiments (Fig. 1c). TI pretreatment could rescue t-BHP-stimulated cell death. (Fig. 1d). In addition, TI reversed t-BHP-induced LDH release in H9c2 cells (Fig. 1e). Simultaneously, apoptosis assay results indicated that TI significantly decreased t-BHP-induced cell death (Fig. 1f, g). All in all, these results indicated that TI exerted protective effects on t-BHP-stimulated H9c2 cells.

### TI ameliorated t-BHP-induced cell necroptosis via RIP1/RIP3/MLKL pathway

**Fig. 2:**
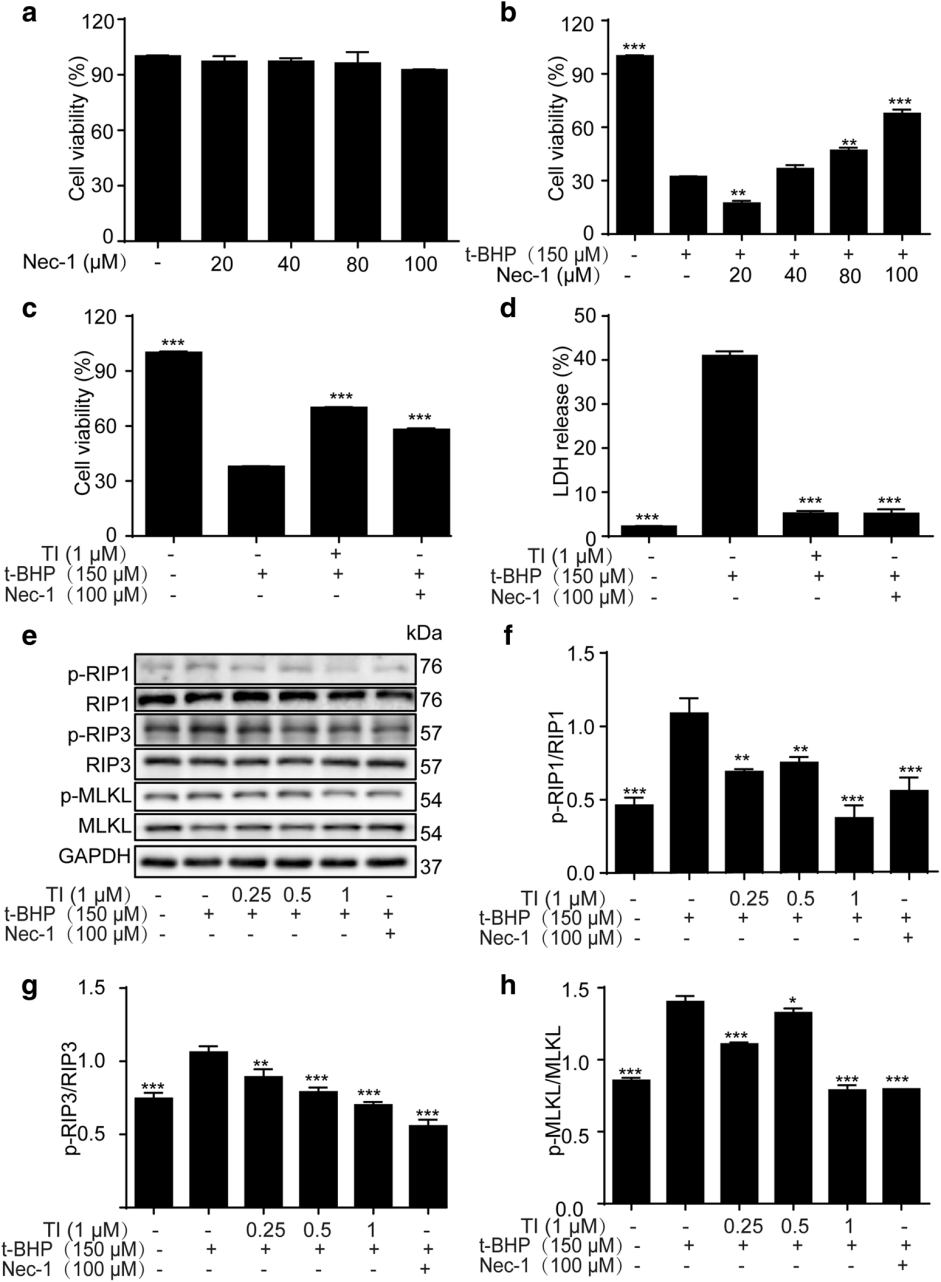
TI ameliorated t‑BHP induced cell necroptosis via RIP1/RIP3/MLKL pathway. **a** H9c2 cells were cultured with Nec-1 for 12 h. **b** Cells were exposed to t-BHP (150 μM) for 10 h after treated with Nec-1 for 2 h. **c** Cells were exposed to t-BHP (150 μM) for 10 h after treated with TI (1 μM) or Nec-1 (100 μM) for 2 h. MTT was employed to detect cell viability. **d** Cells were treated with t-BHP (150 μM) for 10 h when pretreated with TI (1 μM) or Nec-1 (100 μM) for 2 h, the LDH level was monitored by LDH kit. **e–h** Pretreated with TI (0.25, 0.5, and 1 μM) or Nec-1 (100 μM) for 2 h respectively, then exposed to t-BHP (150 μM) for 4 h, the protein expression was determined by western blotting. n = 3. ^*^p < 0.05, ^**^p < 0.01, ^***^p < 0.001 *vs*. t-BHP group

As shown in Fig. 2a, Nec-1 (20–100 μM), the necroptosis inhibitor, had no cytotoxicity in H9c2 cells. Nec-1 could prominently inhibit t-BHP-stimulated H9c2 cells necroptosis, particularly the 100 μM (Fig. 2b). Like the effect of Nec-1, TI significantly inhibited t-BHP-induced necroptosis (Fig. 2c), of which the effects were further confirmed by the LDH results (Fig. 2d). Microscopic images also comfimed this results (Additional file 1: Fig. S1). In addition, necroptosis was primarily mediated by the RIP1/RIP3/MLKL signaling pathway [26]. Compared with the control group, t-BHP augmented phosphorylation of RIP1, RIP3, and MLKL, of which TI could reverse these effects (Fig. 2e–h). Collectively, TI reversed t-BHP-induced necroptosis in H9c2 cells.

### TI mitigated t-BHP-induced ROS generation in H9c2 cells

**Fig. 3:**
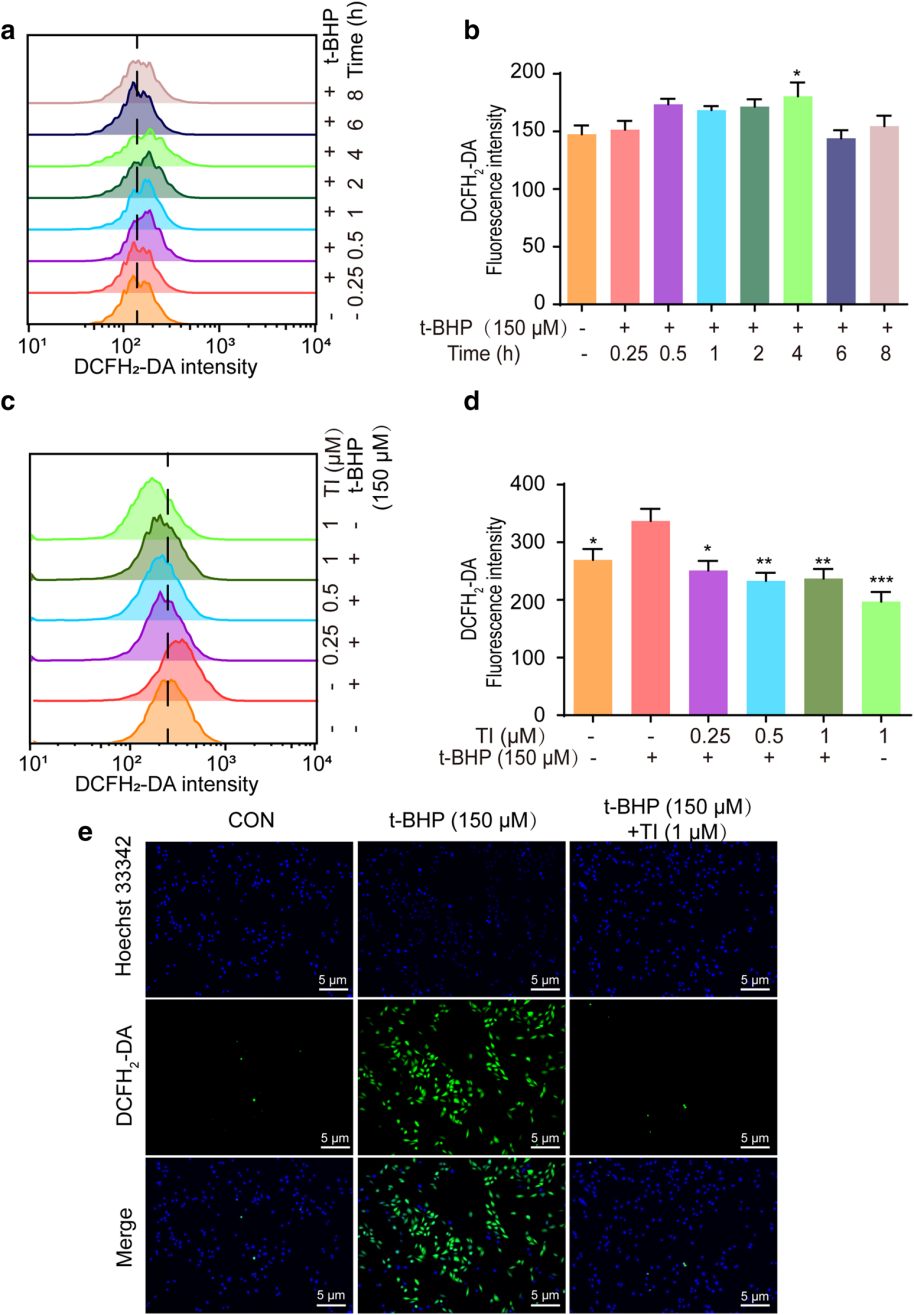
TI mitigated ROS generation in H9c2 cells. **a** H9c2 cells were exposed to t-BHP (150 μM) at different points in time (0, 0.25, 0.5, 1, 2, 4, 6, and 8 h) respectively. Cells labeled with DCFH_2_-DA (1 μM) for 0.5 h were evaluated by flow cytometry. **c** Cells pretreated with TI (1 μM) for 2 h, stimulated with t-BHP (150 μM) for 4 h, then, DCFH_2_-DA (1 μM) was used to stain cells for 0.5 h. The fluorescence intensity was detected by flow cytometry. **b, d** Statistical analysis of the ROS per group. **e** Cells were stained with DCFH_2_-DA for the measurement of ROS level. The images were captured by fluorescence microscopy. n = 3. ^*^p < 0.05, ^**^p < 0.01, ^***^p < 0.00 *vs*. t-BHP group. Scale bar: 5 μm

A sharp augment of ROS level can be stimulated by t-BHP, in the meantime, promoting oxidative stress [27]. The intracellular ROS generation was monitored by immunofluorescent assay and flow cytometry assay. As shown in Fig. 3a, b, t-BHP could augment the ROS level when H9c2 cells were irritated by t-BHP for 4 h. As shown in Fig. 3c–d, TI observably suppressed t-BHP-induced ROS generation in H9c2 cells. In addition, fluorescence microscopy was applied to the observation of ROS production. Results exhibited that TI (1 μM) reduced t-BHP-induced fluorescence intensity dramatically (Fig. 3e), which corresponded to the above results. To sum up, our results revealed that ROS level was lowered by TI, suggesting that TI owned the advantage of anti-oxidative activity.

### TI reversed t-BHP-induced mitochondrial-membrane-potential (MMP) loss

**Fig. 4:**
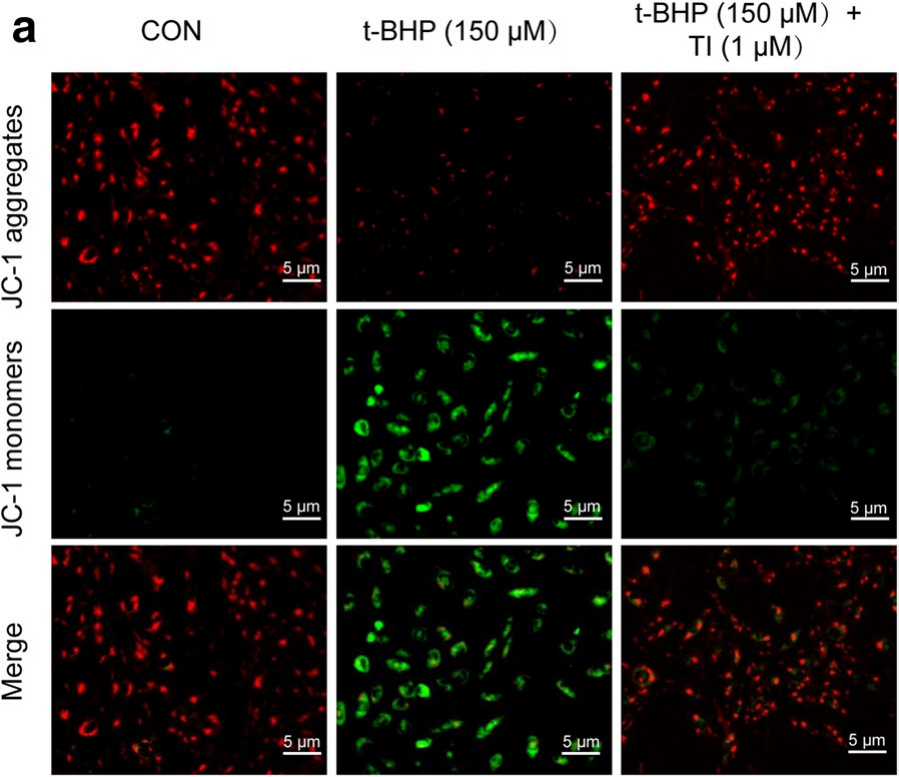
TI could reverse t-BHP-induced MMP loss. **a** Pretreated with TI (1 μM) for 2 h, then exposed to t-BHP (150 μM) for 4 h, cells were then stained with JC-1 (5 μg/mL) for 0.5 h and under a fluorescence microscope, images were captured. Scale bar: 5 μm

t-BHP destroyed the stability of MMP, which was to the disadvantage of keeping normal physiological functions of cells [28]. The changes in MMP were observed by a fluorescence microscope. Results exhibited that under normal circumstances, JC-1 existed in the form of polymer in the cell mitochondria with vivid red fluorescence, and the MMP reduced after cells were treated with t-BHP, which JC-1 could not be present in the mitochondrial matrix as a polymer [29]. Under this circumstance, the red fluorescence’s intensity was markedly decreased, while in the cytoplasm the green fluorescence was signally stronger. This situation was reversed dramatically when the cells were pretreated with TI (1 μM). Collectively, TI (1 μM) rescued t-BHP-stimulated MMP loss (Fig. 4a).

### TI activated Akt/Nrf2 signaling pathway to relieve oxidative stress injury

**Fig. 5:**
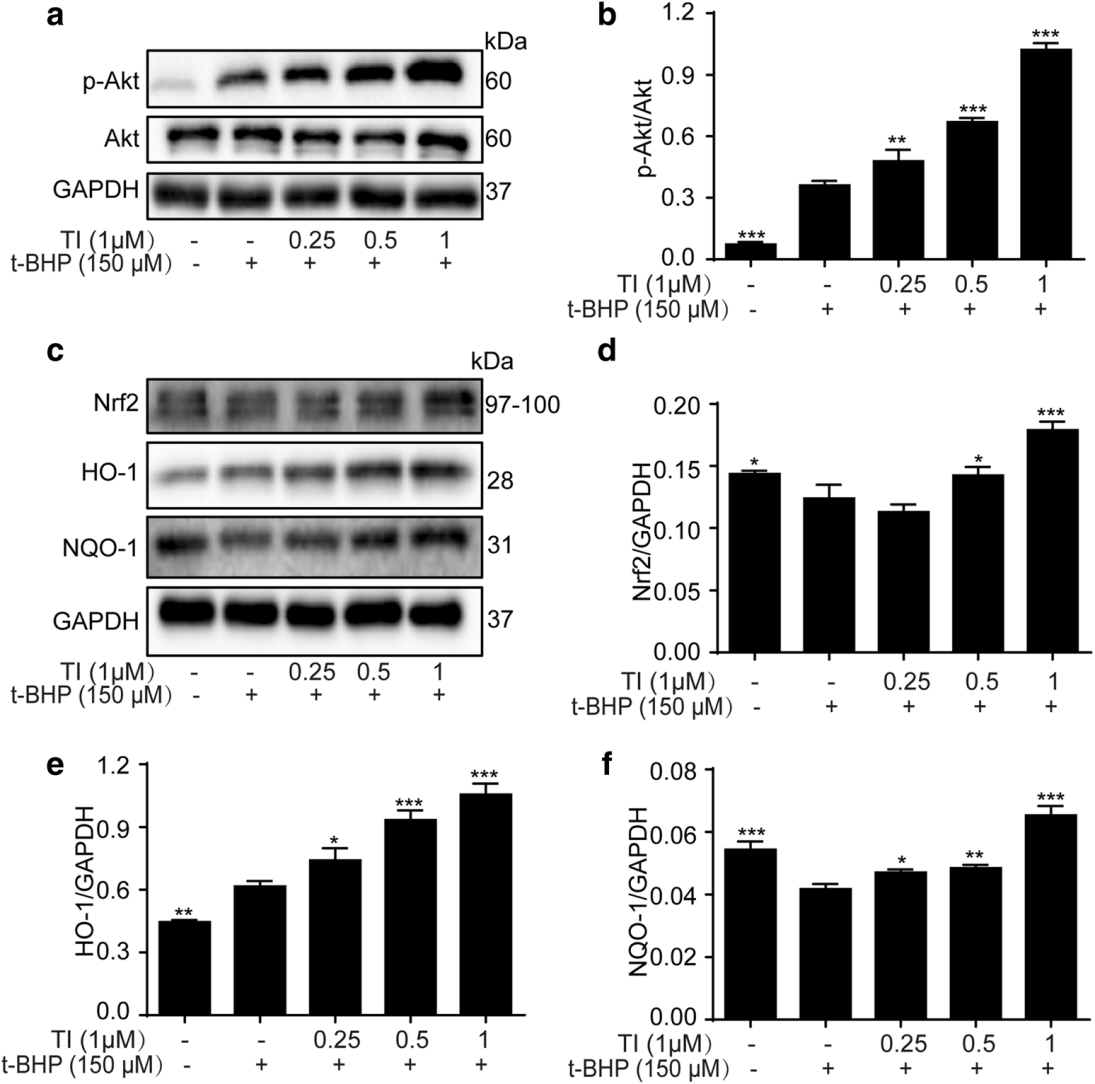
The effect of TI on Akt/Nrf2 pathway. **a, c** H9c2 cells were pretreated with TI (0.25, 0.5, and 1 μM) for 2 h, and exposed to t-BHP (150 μM) for 4 h, therewith, the protein expression were detected by western blotting. Statistical analysis of protein expressions of **b** p-Akt/Akt, **d** Nrf2, **e** HO-1, **f** NQO-1. n = 3. ^*^p < 0.05, ^**^p < 0.01, ^***^p < 0.00 *vs*. t-BHP group

Activation of Akt/Nrf2 signaling pathway occupies a crucial part in the process of anti-oxidative stress [30]. As depicted in Fig. 5a, b, t-BHP lead to the higher expression levels of p-Akt, while TI promoted this effect obviously without altering the Akt. Nrf2, one of the significant nuclear transcription factors, regulates constitutive and inducible expression of anti-oxidant and phase 2 detoxification enzymes coordinately, such as NQO-1 and HO-1 [30, 31]. In the present study, TI markedly increased Nrf2, HO-1, and NQO-1 protein expression. (Fig. 5c–f). In summary, our results demonstrated that TI improved the expression of anti-oxidative-enzyme-related proteins after t-BHP challenge to relieve oxidative stress injury.

### TI pretreatment improved cardiac function and alleviated MI/R injury in SD rats

**Fig. 6:**
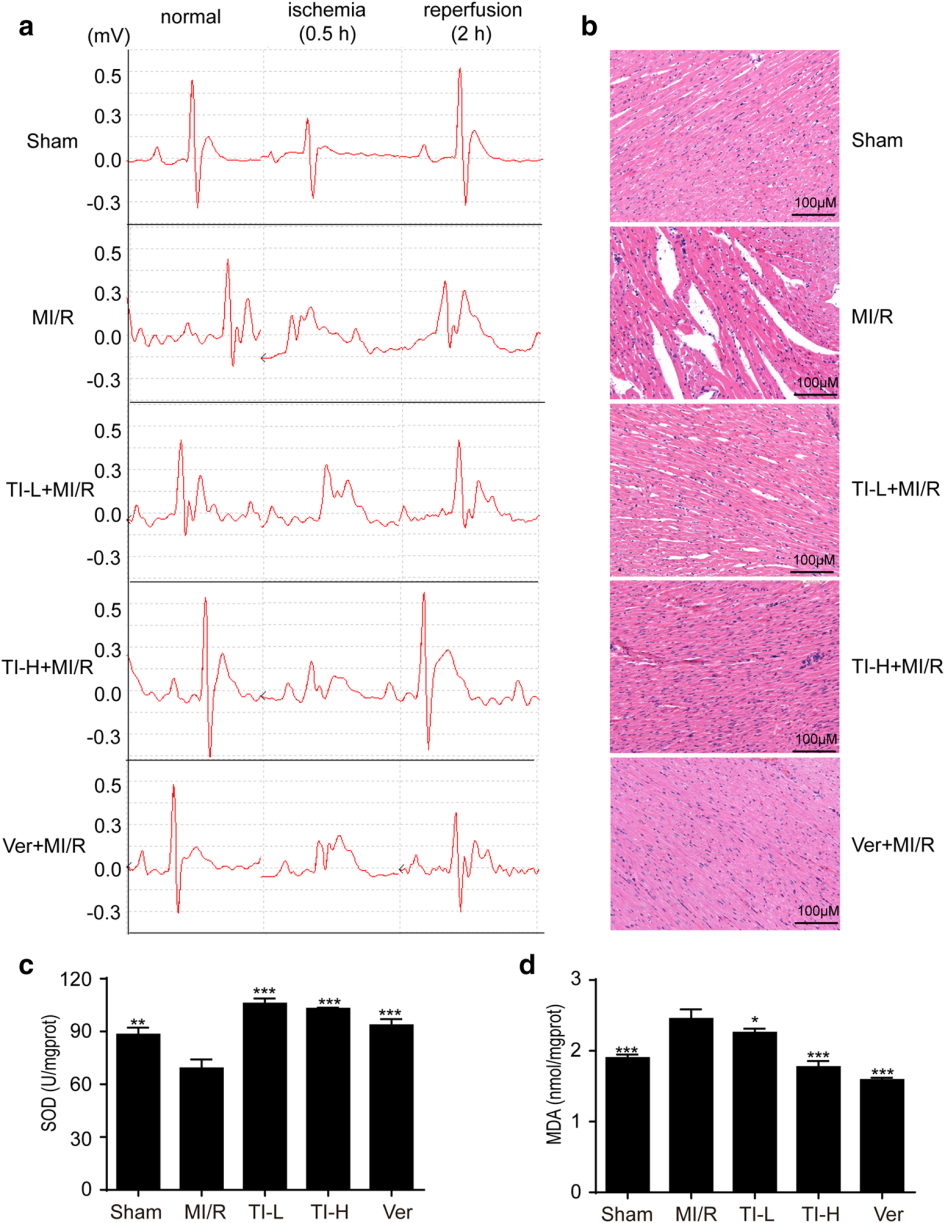
TI pretreatment improved cardiac function and decreased MI/R injury in SD rats. Representative ECG traces in each group (**a**). The degree of myocardial tissue damage during MI/R in rats was assessed by H&E staining (**b**). Hearts were collected and homogenized after ischemia for 30 min and reperfusion for 2 h. The supernatant was collected timely. **c** SOD level and **d** MDA level were investigated by SOD and MDA kits. Value represents mean ± SD (n = 5), *p < 0.05, ***p < 0.001 *vs*. MI/R group. Scale bar: 100 μm

Cardiac function of rats after MI/R injury was detected by ECG. As indicated in Fig. 6a, under normal conditions, TI-treated rats showed no significant difference compared to that of the sham group, suggesting that TI showed no cardiotoxicity. The ST segment of all rats was observably elevated after ischemia, but gradually lowering after reperfusion. Besides, compared with the MI/R group, TI-treatment group and Ver-treatment group’s ECG recovered better after 2 h reperfusion. The H&E staining results revealed that the degree of myocardial tissue damage during MI/R in rats. The myocytes of the sham operation group were normal without bleeding or neutrophil infiltration. Myocardial injury was severe in the ischemic area after MI/R. Myocardial fibers dissolved and infiltrated inflammatory cells. After MI/R, neutrophils congregated in ischemic areas of the heart. TI and Ver treatment alleviated MI/R-induced myocardial injury (Fig. 6b). The level of SOD was reduced by MI/R, but when TI pre-treatment, this change was reversed (Fig. 6c). On the contrary, TI pre-treatment decreased MDA level aggrandized by MI/R in myocardial tissue (Fig. 6d).

### TI restored MI/R-induced imbalance of blood parameters and inhibited inflammatory cytokines release

**Fig. 7:**
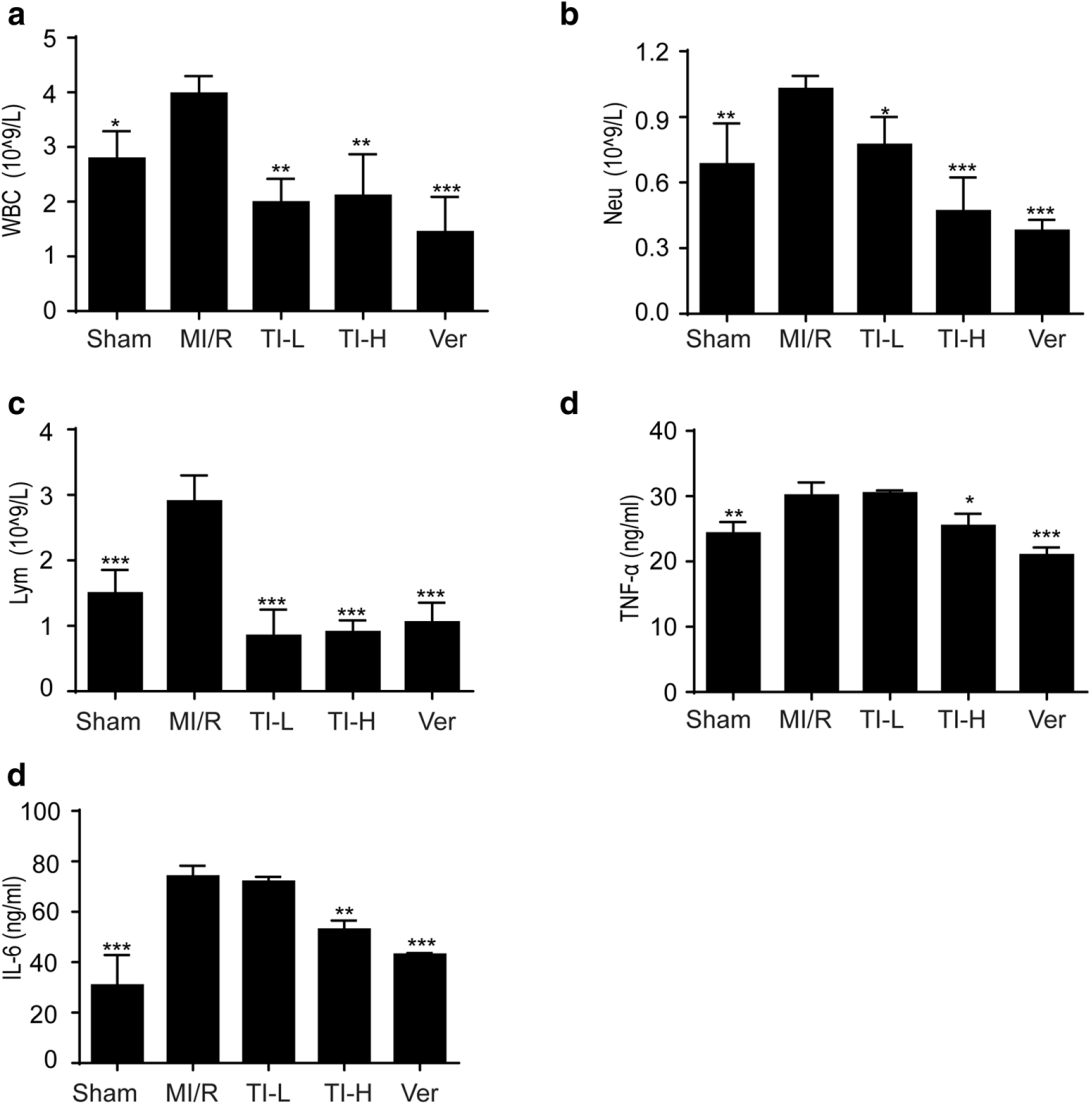
TI pretreatment decreased MI/R injury and inhibited the appearance of inflammation. After 30 min of ischemia and 2 h of reperfusion, blood was collected from the abdominal aorta of rats. **a** Count the amount of WBC. **b** The number of Neu was counted. **c** The counts of lymphocyte (Lym). Hearts were collected, homogenized. The samples were centrifuged (4 °C), the supernatant was collected timely. **d** TNF-α, and **e** IL-6 levels were investigated by ELISA kits following the manufacturer’s instructions. Value represents mean ± SD (n = 5), *p < 0.05, **p < 0.01, ***p < 0.001 *vs*. MI/R group

The blood amount of WBC, Neu, and Lym was the main inflammatory markers detected in routine blood tests [32]. When SD rats encountered MI/R, it caused inflammation. Contrasted with the sham group, the blood counts of WBC (Fig. 7a), Neu (Fig. 7b), and Lym (Fig. 7c) were substantially increased in the MI/R group. However, TI suppressed the abnormal rise of WBC, Neu, as well as Lym, of which its effects amounted to that of the positive drug, Verapamil. Besides, we detected the production of inflammatory cytokines of heart tissue. Compared with the sham operation group, the cytokines like TNF-α and IL-6 levels were augmented after MI/R. In contrast, TI (20 mg/kg) could down-regulate IL-6 and TNF-α levels (Fig. 7d–e).

### TI pretreatment lightened myocardial necroptosis to ameliorate MI/R injury

**Fig. 8:**
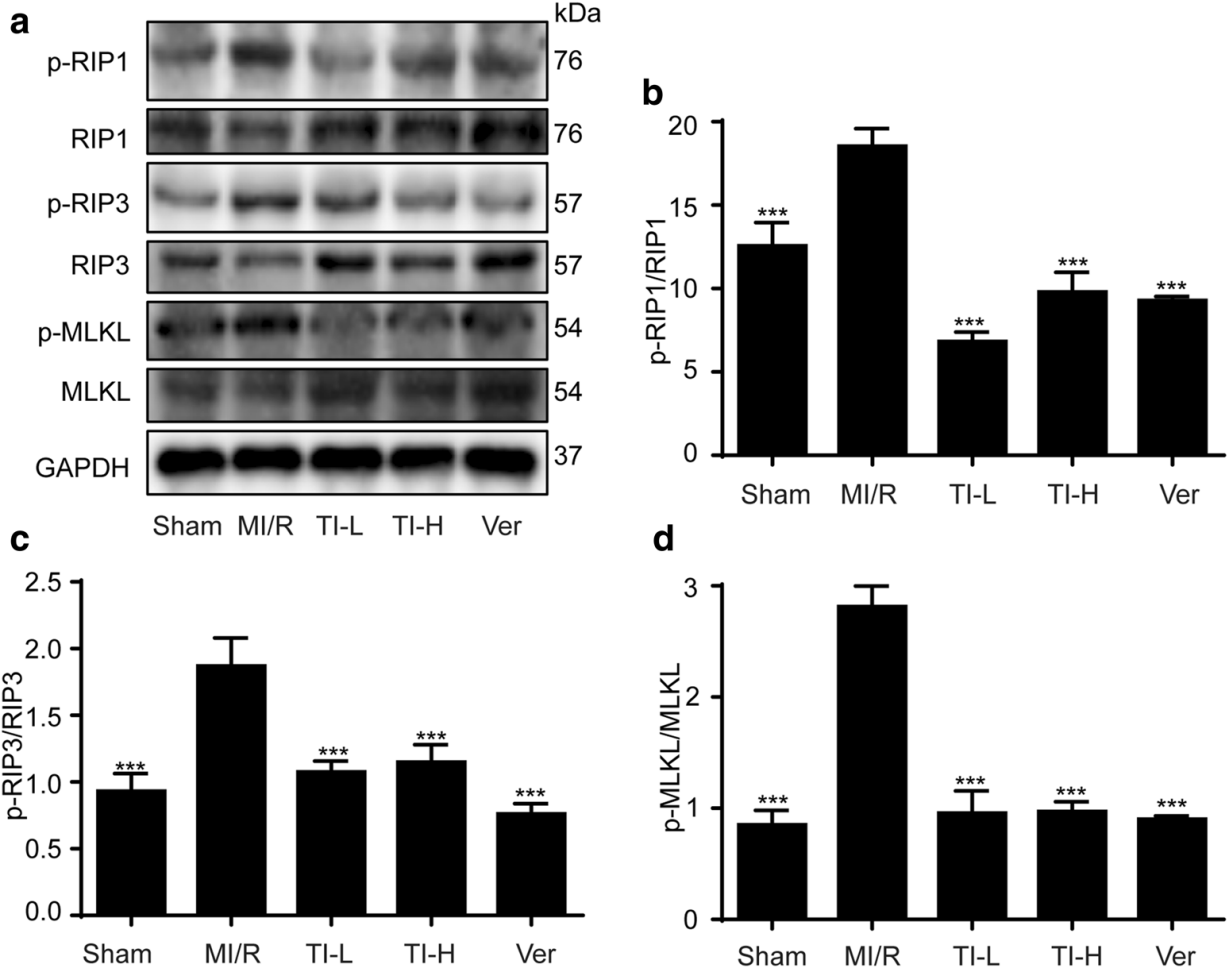
TI pretreatment lightened myocardial necroptosis to improve MI/R injury by RIP1/RIP3/MLKL pathway. After ischemia for 30 min and reperfusion for 2 h, hearts were collected, homogenized. The supernatant was collected to detect the related necroptosis proteins (**a**). The protein expressions of **a, b** p-RIP1/RIP1, **a, c** p-RIP3/RIP3, **a, d** p-MLKL/MLKL were evaluated by western blotting. Value represents mean ± SD (n = 5), **p < 0.01, ***p < 0.001* vs*. MI/R group

The previous study indicated that MI/R injury always led to necroptosis in heart tissues [15]. In our study, using the heart tissues, we found that TI ameliorated MI/R-induced necroptosis. As shown in Fig. 8, TI reversed MI/R-induced the phosphorylation of RIP1, RIP3, and MLKL protein expression.

## Discussion

Cardiovascular disease with high morbidity and mortality is a severe threat to human health [33]. There is increasing evidence that Danshen’s components can prevent cardiovascular diseases, especially atherosclerosis and heart diseases, including myocardial infarction, MI/R injury, arrhythmias, cardiac hypertrophy, and myocardial fibrosis. TI, one of the main fat-soluble components of Danshen, has been used to treat cardiovascular and inflammatory diseases. TI has a protective effect on ischemic injury and can inhibit the proliferation of vascular smooth muscle cells [22, 34]. It also has significant anti-inflammatory activity against both carrageenan-induced foot swelling and adjuvant-induced arthritis [35–38]. In this study, we chiefly focused on the cardiovascular protective effects of TI in vitro and in vivo.

The imbalance of cell death promotes the pathogenesis of cardiovascular diseases and is a clinical target therapy in the course of disease occurrence [14, 39]. Necrosis, previously considered as an unregulated form of cell death, has been recognized as a highly regulated process up to now and has attracted widespread attention in the past decade [39, 40]. In addition, necroptosis, a kind of programmed necrosis, would be exacerbated in t-BHP-induced endothelial cells [28]. Recent evidence showed that a significant increase in intracellular ROS results in cell necroptosis [41, 42]. ROS is involved in TNF-induced necroptosis of human colon adenocarcinoma HT-29 cells [41]. t-BHP, a better alternative for unstable H_2_O_2_, is an organic peroxide broadly used in multiple oxidative stress studies and can liberate ROS, resulting in oxidative stress injury [43]. Whereas still much less is known about the contribution of t-BHP- stimulated oxidative stress injury and necroptosis in H9c2 cells. Furthermore, it has been reported that enhanced Akt phosphorylation promotes cardiac myocyte survival [19, 44]. And Nrf2 is normally deemed to be a key transcription factor in anti-oxidative stress by strengthening antioxidant genes’ expression such as HO-1 and NQO-1. In this study, we found that TI could conspicuously decrease ROS generation (Fig. 3a–e) and relieve oxidative stress injury through activating Akt/Nrf2 signaling pathways, promoting the phosphorylation of Akt, and the expression of anti-oxidative-enzyme-related proteins, namely Nrf2, NQO-1 and HO-1(Fig. 5a–f). Even superfluous ROS impacted mitochondria performance by decreasing MMP negatively [45], which was mitigated by TI (1 μM) (Fig. 4a).

MI/R injury is an urgent problem in clinical treatment [46]. MI/R injury irreversibly damage the structure and function of the heart [4]. With the in-depth study on the anti-MI/R injury efficacy of traditional Chinese medicine, it has been confirmed that it can alleviate MI/R injury by regulating programmed necrosis [47]. TNFR1-induced necroptosis is incurred by a signaling cascade referring to special serine-threonine proteins RIP1, RIP3, and MLKL [48]. Nevertheless, potential therapeutic reagents, such as Nec-1 inhibitor, could offer protection from the adverse effects of necroptosis [49]. Hence, our study focused on the relation between MI/R and necroptosis. Gratifyingly, TI conferred a favorable role in the improvement of MI/R injury via restraining p-RIP1, p-RIP3, and p-MLKL expression (Fig. 8a–d).

Under physiological conditions, ROS acts as a second messenger to regulate a variety of signaling pathways [50]. Nonetheless, excess intracellular ROS leads to damage to the molecular components of cells, contributing to a wide variety of pathogenesis human diseases. A large number of studies have shown that ROS production is closely related to the process of cell death and promotes the occurrence of cell death as well as the development of cardiovascular diseases. Studies have shown that when myocardial ischemia, blood recovers after hypoxia, and oxygen supply will produce overmuch ROS, which will culminate in oxidative stress in the myocardium induced MI/R injury [51]. Electron paramagnetic resonance (EPR) was employed to directly measure myocardial free radicals and it was found that a large amount of ROS was produced immediately after MI/R, which lasted for tens of minutes [52]. Fortunately, SOD is the main antioxidant enzyme that removes oxidative free radicals in the body, and its activity can reflect the ability to reduce oxidative free radicals. When ROS reacts with a polyunsaturated fatty acid, its lipid peroxide products, such as MDA, can reflect its lipid peroxide damage and indirectly indicate the injury of ROS to the body [53, 54]. Our research elucidated that the level of SOD was reduced by MI/R in contrast with sham operation group and this effect was reversed by TI pre-treatment (Fig. 6c). Oppositely, the level of MDA in heart tissue was aggrandized by MI/R and TI pre-treatment observably decrease this effect (Fig. 6d). which manifested the anti-oxidative properties of TI.

Of note, not only does the generation of ROS mediate the above aspects, but also it promotes inflammatory response [55]. Inflammatory response is the main cause of further myocardial damage and dysfunction. More and more evidences show that a large number of inflammatory mediators and chemokines are produced in the process of MI/R injury. Activated neutrophils can secrete cytokines, such as TNF-α and IL-6, which can further damage the myocardium, leading to metabolic dysfunction, degeneration and necrosis, etc. [56–59]. TNF-α is a crucial pro-inflammatory cytokine, which exerts key effects in the synthesis and release of inflammatory mediators, complement activation, and other physiological responses [38]. A pivotal cytokine, IL-6, is produced by fibroblasts as well as T cells, which are in a state of activation, and occupy comprehensive biological activities, for instance, immune regulation [60, 61]. Accumulating evidence has indicated that not only can IL-6 catalyze inflammatory response, but also it can amplify it. Its level of expression is able to reflect the seriousness of tissue cell injury effectually, and IL-6 can be utilized as a valid index for chronic as well as acute inflammation in terms of clinical diagnosis [62]. Our study showed that TI could prevent myocardial fibers dissolved and infiltrated inflammatory cells (Fig. 6b). TI lowered the amount of WBC, Neu and Lym in blood of rats encountering MI/R (Fig. 7a–c). Besides, TI exerted significant inhibitory effects on TNF-α and IL-6 (Fig. 7d, e). Therefore, TI owned a prominently protective effect on assuaging MI/R injury.

## Conclusion

**Fig. 9:**
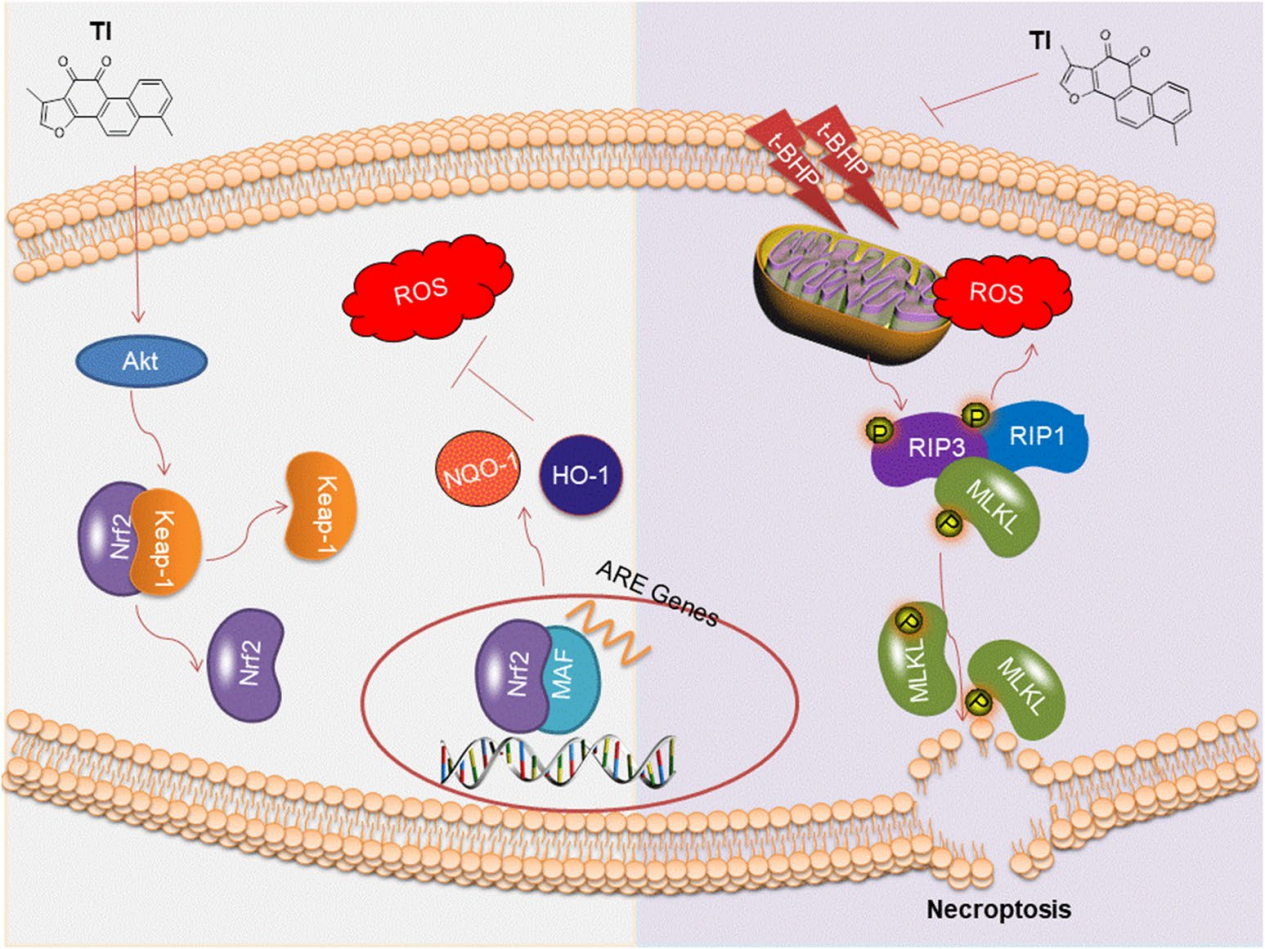
The schematic of cardiovascular protective effects of TI in vitro. The mechanism of TI’s anti-oxidative effects and its effect on alleviating necroptosis in vitro was through Akt/Nrf2 and RIP1/RIP3/MLKL signaling pathways

To sum up, our data indicated that TI could relieve t-BHP-stimulated oxidative stress injury by suppressing RIP1/RIP3/MLKL and activating Akt/Nrf2 signaling pathways (As shown in Fig. 9). Also, TI could improve cardiac function in response to MI/R injury by regulation of RIP1/RIP3/MLKL signaling pathway. These underlying mechanisms of TI highlighted that TI owned the advantage of promising cardiovascular protection activities, which facilitated the development of a novel agent for the treatment of cardiovascular diseases.

## Supplementary Information

**Additional file 1: Fig. S1** The detection of cells’ morphological changes. H9c2 cells were pretreated with TI (1 μM) or nec-1 (100 μM)for 2 h, then exposed to t-BHP (150 μM) for 6 h, the cellular morphology was detected by microscope (Olympus, Tokyo, Japan).

## Data Availability

Not applicable.

## Abbreviations

TI: Tanshinone I
t-BHP: Tert-butylhydroperoxide
Ver: Verapamil hydrochloride
MTT: 3-(4,5-Dimethylthiazol-2-yl)-2,5-diphenyl tetrazolium bromide
LDH: Lactate dehydrogenase
ROS: Reactive-oxygen-species
MMP: Mitochondrial membrane potential
MI/R: Myocardial ischemia–reperfusion
H&E: Hematoxylin–eosin
SOD: Superoxide dismutase
MDA: Malondialdehyde
TNF-α: Tumor necrosis factor-α
IL-6: Interleukin-6
RIP1: Receptor interacting protein kinase 1
RIP3: Receptor interacting protein kinase 3
MLKL: Mixed lineage kinase domain-like protein
WBC: White blood cell
Neu: Neutrophils
Lym: Lymphocyte
DMEM: Dulbecco’s modified eagle medium
DMSO: Dimethylsulfoxide
ELISA: Enzyme-linked immunosorbent assay
FBS: Fetal bovine serum
Nrf2: Nuclear factor erythroid 2 related factor 2
HO-1: Heme oxygenase-1
NQO-1: NAD(P)H: quinone oxidoreductase-1
PVDF: Polyvinylidene fluoride
ECG: Electrocardiograph
EPR: Electron paramagnetic resonance
LAD: Left anterior descending coronary artery
