# Supplementary figures and images for: Tanshinone I exerts cardiovascular protective effects in vivo and in vitro through inhibiting necroptosis via Akt/Nrf2 signaling pathway
Source: Chin Med. 2021 Jun 28;16:48. doi: 10.1186/s13020-021-00458-7 (PMC8240219; doi:10.1186/s13020-021-00458-7)

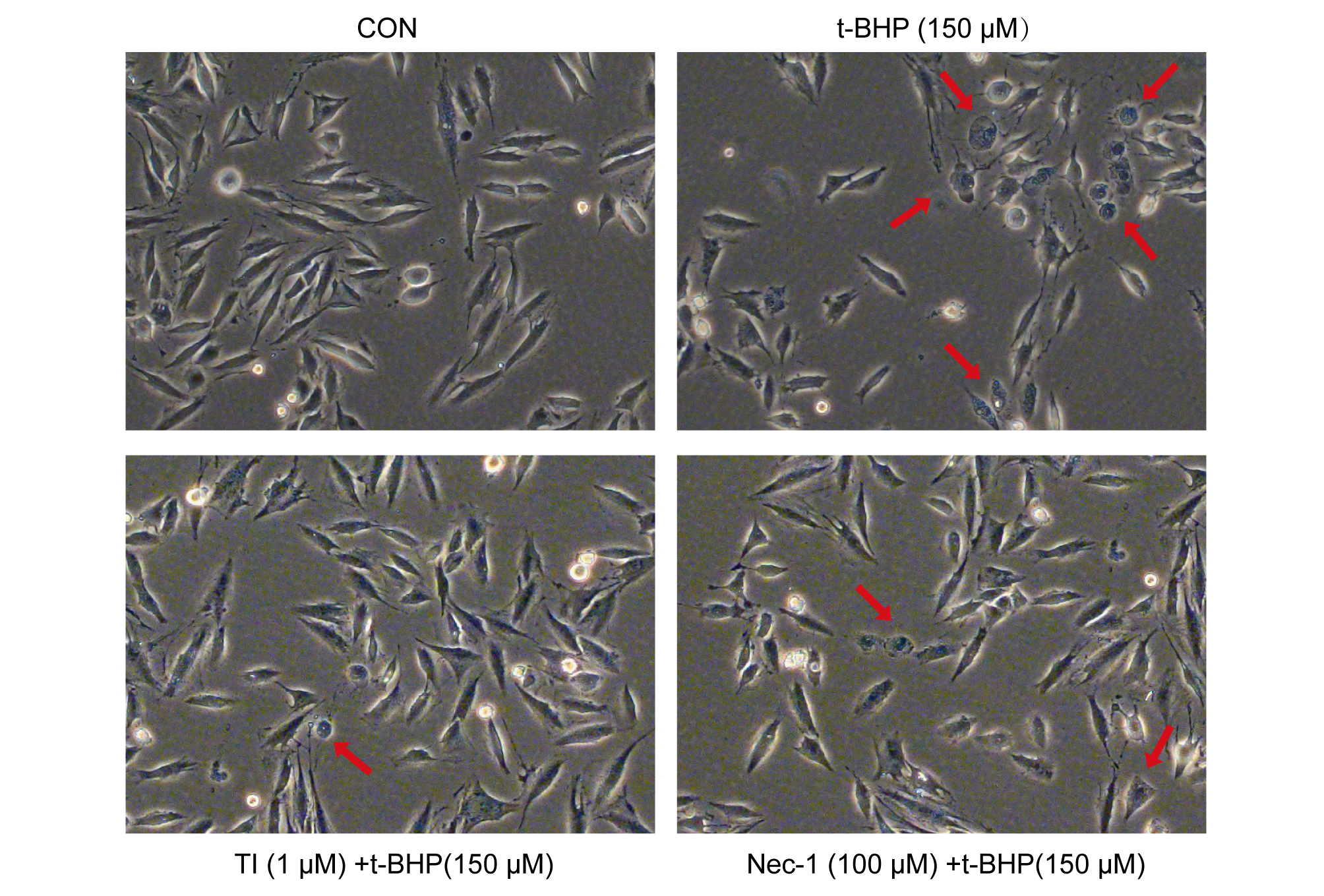

Supplement: Supplementary file 1 — Additional file 1: Fig. S1 The detection of cells’ morphological changes. H9c2 cells were pretreated with TI (1 μM) or nec-1 (100 μM)for 2 h, then exposed to t-BHP (150 μM) for 6 h, the cellular morphology was detected by microscope (Olympus, Tokyo, Japan). [file 13020_2021_458_MOESM1_ESM.tif]
